# *In actio* optophysiological analyses reveal functional diversification of dopaminergic neurons in the nematode *C. elegans*

**DOI:** 10.1038/srep26297

**Published:** 2016-05-19

**Authors:** Yuki Tanimoto, Ying Grace Zheng, Xianfeng Fei, Yukako Fujie, Koichi Hashimoto, Koutarou D. Kimura

**Affiliations:** 1 Department of Biological Sciences, Graduate School of Science, Osaka University, Toyonaka, Osaka 560-0043, Japan; 2 Faculty of Science and Technology, Tohoku Bunka Gakuen Univ., Sendai 981-0943, Japan; 3 Graduate School of Information Sciences, Tohoku University, Sendai, Miyagi 980-8579, Japan

## Abstract

Many neuronal groups such as dopamine-releasing (dopaminergic) neurons are functionally divergent, although the details of such divergence are not well understood. Dopamine in the nematode *Caenorhabditis elegans* modulates various neural functions and is released from four left-right pairs of neurons. The terminal identities of these dopaminergic neurons are regulated by the same genetic program, and previous studies have suggested that they are functionally redundant. In this study, however, we show functional divergence within the dopaminergic neurons of *C. elegans*. Because dopaminergic neurons of the animals were supposedly activated by mechanical stimulus upon entry into a lawn of their food bacteria, we developed a novel integrated microscope system that can auto-track a freely-moving (*in actio*) *C. elegans* to individually monitor and stimulate the neuronal activities of multiple neurons. We found that only head-dorsal pair of dopaminergic neurons (CEPD), but not head-ventral or posterior pairs, were preferentially activated upon food entry. In addition, the optogenetic activation of CEPD neurons alone exhibited effects similar to those observed upon food entry. Thus, our results demonstrated functional divergence in the genetically similar dopaminergic neurons, which may provide a new entry point toward understanding functional diversity of neurons beyond genetic terminal identification.

A variety of neuronal types exist in an animal’s brain, each of which plays specific physiological roles based on its own property for integrative brain function. Neuronal cell types are distinguished primarily on the basis of differences in gene expression as well as anatomical properties such as localization, morphology, and interneuronal connectivity. These divergences are regulated by genetic programs as well as temporal and positional interactions^1^,^2^.

Dopamine (DA) is a neurotransmitter that has been investigated intensively owing to its prominent roles in the brain functions of higher animals. It is responsible for locomotor regulation, cognition, emotion, reward, and learning, and its dysfunction causes Parkinson’s disease, schizophrenia, and drug addiction^3^. Despite having such extensive roles, interestingly, the cell bodies of dopaminergic (DAergic) neurons are located in limited areas of the brain such as the midbrain and hypothalamus^4^. Recent studies have revealed the functional divergence of DAergic neurons, such as differences in sensory information to which they respond and their response patterns^5^,^6^,^7^. Nonetheless, the detailed properties and mechanisms of such divergence have not been sufficiently elucidated.

Likewise, DA is released from a limited number of neurons and regulates a variety of neural functions, such as locomotion, sensory perception, and learning, in the nematode *Caenorhabditis elegans*^8^,^9^,^10^,^11^,^12^,^13^,^14^,^15^. Leveraging the animal’s advantages, such as its small nervous system consisting of just 302 neurons, feasibility of genetic analyses, and utility of a series of cell-specific promoters^16^,^17^, the regulatory mechanisms of DA signalling on these neural functions have been studied at the cellular and molecular levels. In hermaphrodites (the major sexual form of the animals), four left-right pairs of neurons are DAergic: CEPD (cephalic sensilla-dorsal), CEPV (cephalic sensilla-ventral), ADE (anterior deirids), and PDE (posterior deirids)^18^ (Fig. 1a; note that the animals’ left-right axis is vertical and their dorsal-ventral axis is horizontal when they move in the standard laboratory condition). The terminal identities of all of these DAergic neurons are tightly regulated by the same *cis*-regulatory elements and the same transcription factors in *C. elegans* as in mammals^19^,^20^. In addition, D1- and D2-type receptors function in multiple types of neurons of the animals^21^, a phenomenon that is analogous to DA signalling in mammals.

Previous studies have suggested that DAergic neurons in *C. elegans* are activated by the presence of their food bacteria, one of the most influential environmental stimuli modulating diverse aspects of their behaviour. For instance, the animal’s locomotor speed is slowed upon entry into a bacterial lawn, which is mimicked by a mechanical stimulus from Sephadex beads^8^. This slowing response is mediated by DA signalling, and laser ablation of all four pairs DAergic neurons was necessary to fully suppress the slowing, suggesting that the neurons function redundantly^8^. Recently, the use of a genetically encoded calcium indicator^22^ revealed the head DAergic CEP pairs were activated by mechanical stimulation with a glass tip^12^. Moreover, optogenetic stimulation^23^,^24^ of all DAergic neurons caused slowing^25^. However, the actual relationship between bacterial presence and DAergic neuron activities remains unclear. Could the bacterial lawn provide a sufficient mechanical stimulus to activate DAergic neurons? In what manner (tonically or phasically, for example) are DAergic neurons activated? Which DAergic neurons are indeed activated by food entry? These questions should be answered to understand how food presence, the most influential information for the animals, is transformed into sensory neural activity and then transmitted to downstream target neurons to modulate multiple behavioural aspects. However, the methodology for studying this relationship has been difficult. For calcium imaging, since the bacterial presence is sensed mechanically but not chemically, the widely used microfluidic flow channel for animal’s stimulation^26^ is not applicable. Although auto-tracking microscope systems for *in vivo* calcium imaging or optogenetic stimulation of freely-moving *C. elegans* recently became available^27^,^28^,^29^,^30^,^31^,^32^, separately analysing the activities of individual DAergic neurons remains challenging because 3 pairs of DAergic neurons (CEPD, CEPV, and ADE) are all located in the head ganglia (Fig. 1a), and the machine-vision discrimination of these neurons has been difficult, especially during tracking.

Here we established an integrated microscope system for calcium imaging and optogenetic stimulation of individual neurons of freely-moving (*in actio*) *C. elegans* with high spatiotemporal resolution. While the terminal identities of all of the DAergic neurons are tightly regulated by the same genetic program^19^,^20^ and these neurons are considered functionally redundant^8^, we found functional diversification among DAergic neuron pairs in the slowing response upon food entry. In particular, the CEPD neurons were preferentially activated upon food entry compared to the CEPV neurons despite their symmetry at the gross anatomical level^33^ (Fig. 1a). The posterior PDE neurons did not appear to be activated. Moreover, the optogenetic activation of CEPD neurons, but not of CEPV or PDE neurons, caused a behavioural response similar to that of food entry. Thus, our results demonstrate the functional diversification of genetically and even anatomically similar DAergic neurons.

## Results

### Experimental setup for optophysiological analysis during food entry

**Figure 1:**
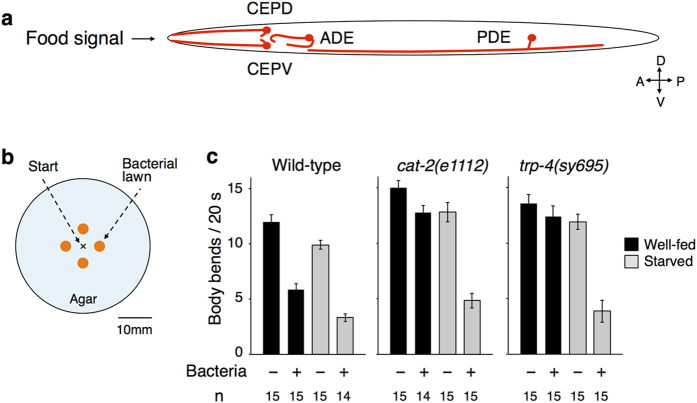
DAergic neurons mediate food-dependent slowing behaviour. (**a**) A schematic diagram of arrangement of the four DAergic neuron pairs. Note that this is the top view of an animal on an agar surface. (**b**) An arrangement of patches of bacterial lawns on an agar surface. Similar experimental condition was recently reported by Hardaway *et al*.^38^. (**c**) Food-dependent slowing response of wild-type and mutant animals. Well-fed animals (black bars) or animals starved for 30 min (grey bars) were transferred to the centre position of the assay plate shown in *b* and the bending numbers were scored for 20 s after the food entry. The numbers of animals used in each condition are shown at the bottom.

Here we focused on the slowing of locomotion upon the entry of a food bacterial lawn^8^, one of the most prominent DA-dependent behavioural responses of *C. elegans*. The animals reduces the frequency of its body bending and slows down its locomotion upon food entry, which is likely because the animals migrate more quickly to find out food when they are off food, and they migrate more slowly on food to consume it. For the optophysiological analysis of the slowing response upon food entry, we optimized the experimental condition (Fig. 1b). Even with a thin layer (30–40 μm) of bacterial lawn that does not disturb fluorescent imaging, the food entry–dependent slowing response was reproduced (Fig. 1c). The slowing response was affected by mutations in tyrosine hydroxylase *cat-2* or TRPN channel *trp-4*, which are required for DA biosynthesis or DAergic neuron activation, respectively, as previously reported^8^,^12^ (Fig. 1c, black bars). The slowing response was observed in both *cat-2* and *trp-4* mutants after 30 min of starvation (Fig. 1c, grey bars), which is consistent with the fact that 30-min starvation causes an enhanced slowing response that is dependent on serotonin, and not on dopamine^8^.

### Cell-targeted calcium imaging and optogenetics system for freely-moving animals

**Figure 2:**
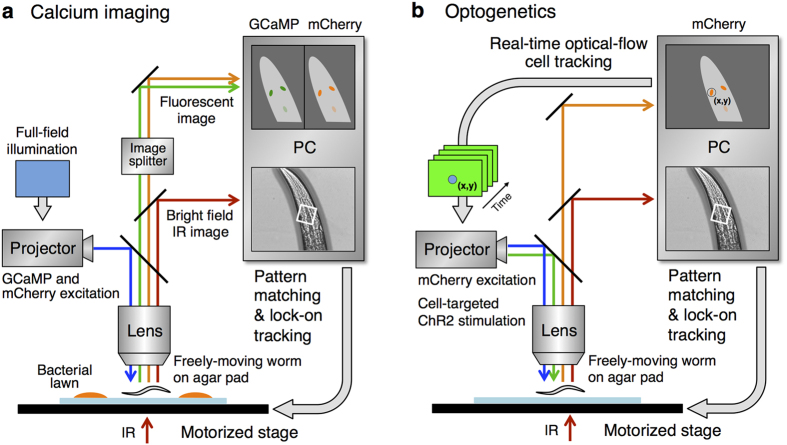
Schematic drawing of the OSaCaBeN system. Setups for calcium imaging (**a**) and optogenetic analysis (**b**) are shown. The motorized stage is controlled to lock-on a part of the animal’s body at the centre of the view field. In (**a**), blue light is illuminated to the full view field to excite GCaMP6f and mCherry in all of the DAergic neurons. In (**b**), green light for mCherry is illuminated to the full view field to monitor the positions of all of the DAergic neurons, and blue light is illuminated to one of the DAergic neurons to stimulate ChR2 when necessary. The position of the blue light illumination is updated in real time because the target neuron moves in the view field due to the limitation of tracking accuracy. An IR light was used to acquire bright field images for pattern matching and tracking.

To optophysiologically analyse multiple DA neuron pairs *in actio*, we developed an auto-tracking microscope system featuring sophisticated image processing and machine-control techniques (Fig. 2 and Supplementary Video S1). The auto-tracking system maintains the head of a freely-moving *C. elegans* in the centre of the field of view using a texture pattern-matching algorithm^34^.

In the calcium imaging setup, the images of green fluorescence from GCaMP6f 35 and red fluorescence from mCherry^36^ were used to monitor the intracellular calcium concentration changes and the cell body positions of the multiple DAergic neurons, respectively. The individual signal intensities at each of the cell bodies were measured separately off-line. However, the conventional method that tracks the brightest point in the view field is not applicable. Thus, here we developed a software that individually tracks multiple fluorescent signals using the optical flow method (Supplementary Video S2). It should be noted that the measured fluorescent signal from one cell body likely reflects the sum of vertically aligned a pair of left and right neurons.

In the optogenetics setup, the optical flow method is used on-line: channelrhodopsin-2 (ChR2), a light-gated cation channel^23^ and mCherry are co-expressed in the DAergic neurons, and the mCherry signals were used to identify the positions of multiple DAergic neuron pairs. Blue light is illuminated only at the instantaneous position of the targeted cell using a high power video projector as a light source^37^ (Supplementary Video S3). With the high speed of its tracking system, this method allows for optogenetic activation of one among multiple target cells in the head ganglia of the moving *C. elegans* (see Discussion). We named this the *O*ptogenetic *S*timulation *a*ssociated with *Ca*lcium imaging for *Be*having *N*ematodes (OSaCaBeN or OSB) system.

### Anatomically symmetrical CEPD and CEPV neurons are asymmetrically activated

**Figure 3:**
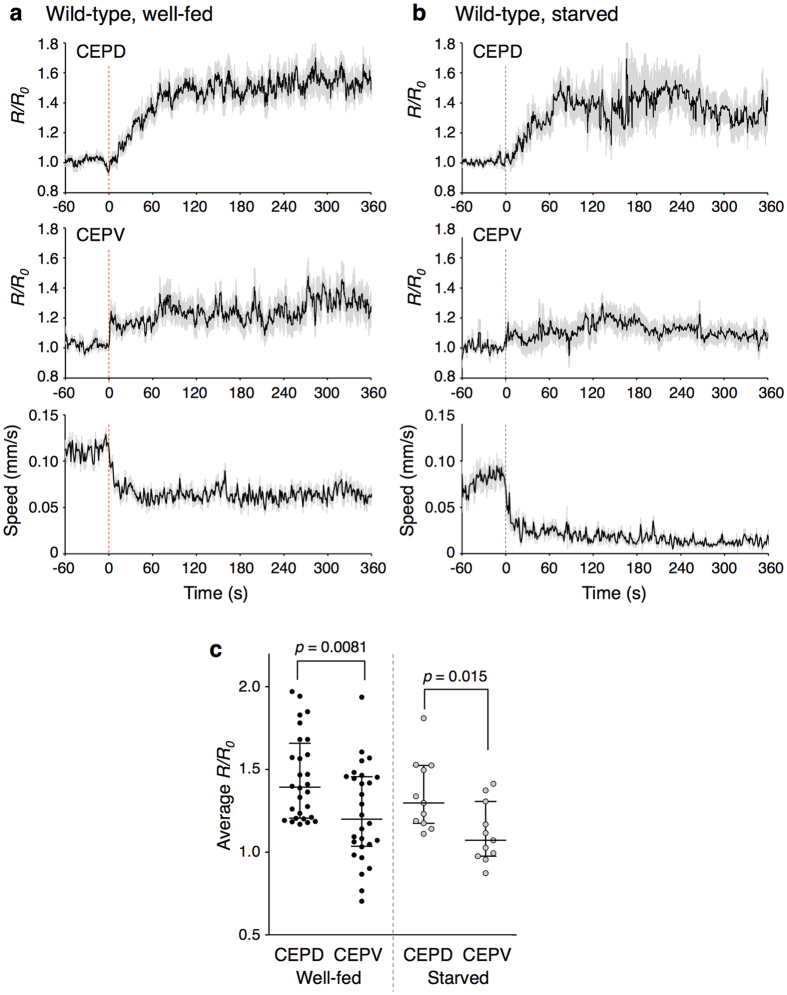
CEPD and CEPV neuron pairs were asymmetrically activated upon food entry. (**a,b**) Responses of CEPD (top) and CEPV (middle) as well as speed (bottom) of well-fed (**a**) or starved (**b**) wild-type animals upon food entry are shown. The time when an animal entered a bacterial lawn was determined as *t* = 0. The *R/R*_*0*_ values (average ± SEM) are shown. The responses of CEPD and CEPV were monitored from the same animals. Twenty-eight and 11 animals were analysed for well-fed and starved conditions, respectively. When the average *R/R*_*0*_ value was lower than 1.1 in both of CEPD and CEPV in an animal, that animal was regarded as “not responding”, and those data were excluded. (**c**) Scatter plot comparing averaged *R/R*_*0*_ values between CEPD and CEPV of well-fed (left) or starved (right) wild-type animals upon food entry. A two-tailed Mann-Whitney test was used for the statistical analysis.

Using the calcium imaging setup, we measured the activities of the CEPD and CEPV neurons, which are symmetrical in the dorso-ventral axis at the gross anatomical level and extend their sensory endings to the anterior end of the body^33^ (Fig. 1a). Upon food entry, which likely produces a subtle mechanical force, CEPD and CEPV pairs were activated as reported recently^38^, and the activity was sustained at least for 6 minutes (Fig. 3a). Unexpectedly, however, we found that the CEPD activation was gradual and stronger, while CEPV activation was quicker and weaker (Fig. 3a,c). These results indicate that CEPD and CEPV pair activities upon food entry are asymmetrical, although these neuron pairs are considered genetically indistinguishable and functionally redundant. We also analysed the PDE pair of DAergic neurons, whose processes cover more than half of the posterior end of the body (Fig. 1a). Food entry-dependent activation of PDE pair was observed only in a few trials (Table 1, Fig. S1). The ADE neuron pair could not be analysed because of dim GCaMP fluorescence from the cells, possibly due to low intracellular calcium concentration before and after food entry. These results suggest that CEPD and CEPV neuron pairs sense the bacteria presence upon food entry, whereas ADE and PDE neuron pairs may be devoted to a different role. In *trp-4* mutants, CEPD and CEPV showed little response upon food entry (Table 1, Fig. S1). This result is consistent with the fact that CEP neurons of the *trp-4* mutant are defective in response to a certain type of mechanical stimulus: *trp-4* mutants did not respond to gentle and repetitive press stimulus, but responded to a continuous press^12^.

We further measured CEPD and CEPV pair activities under starved condition. Starvation is known to modulate various aspects of *C. elegans’* behaviour^16^,^17^, and DA-dependent behavioural modulations such as area-restricted search and tap habituation are generally not observable after 30 minutes of starvation^10^,^12^. In addition, after starvation, the slowing response upon food entry is enhanced in a serotonin-dependent manner^8^ (Fig. 1c). Regardless, it was not clear whether the responses of DAergic neurons themselves are modulated by starvation. We found the responses of CEPD and CEPV neurons to bacterial lawn were essentially similar before and after starvation (Fig. 3b,c), while the slowing response was enhanced (Fig. 3a,b, bottom). These results suggest that starvation-induced modulation of DA signalling may occur not in the DAergic neurons themselves but rather in the target neurons, such as sensory or motor neurons^12^,^25^,^39^.

### CEPD is mainly responsible for food-dependent slowing behaviour

**Figure 4:**
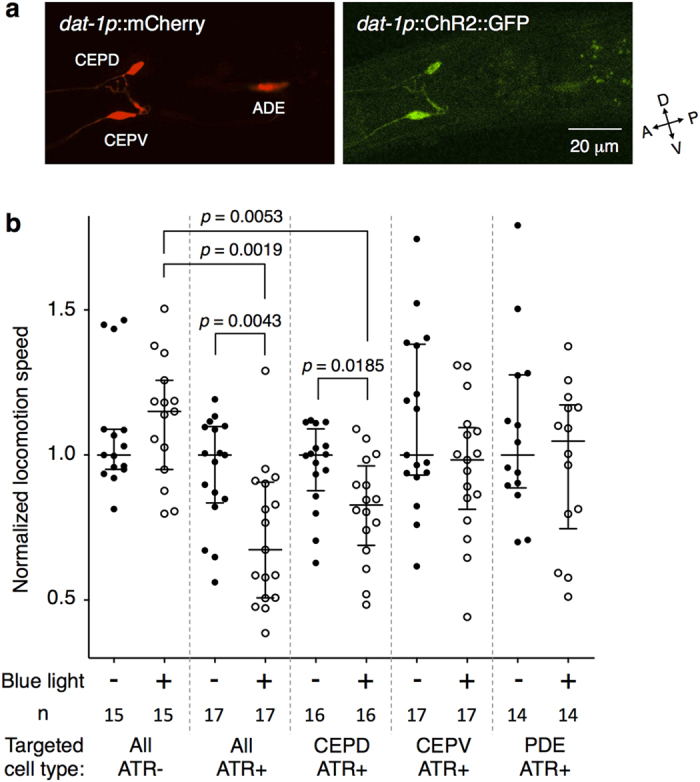
Activation of CEPD neuron pairs is mainly responsible for the slowing behaviour. (**a**) Expressions of mCherry and ChR2::GFP were at similar levels between CEPD and CEPV but was lower in ADE. (**b**) Comparison of the effects of optogenetic stimulation on slowing. When all of the dopaminergic neurons or only CEPD were illuminated, but not CEPV or PDE, significant slowing occurred. A Mann-Whitney test was used to compare the normalized locomotion speeds before (−) and during (+) the blue light illumination within each targeted cell type, while a Kruskal-Wallis test with a *post-hoc* Steel-Dwass test was used to compare cell types during illumination. Details of the statistical analyses are shown in Supplementary Table S4.

Calcium imaging revealed that CEPD and CEPV pairs were activated asymmetrically upon food entry. However, the relationship between this asymmetrical activation and their behavioural consequences is still unclear. To further elucidate the functional differences in DAergic neuron activities, we optogenetically activated the individual DAergic neuron pairs and analysed the effects on the slowing response. We established transgenic animals expressing ChR2 and mCherry in all of the DAergic neurons at similar levels in the cells (Fig. 4a). Blue light illumination to the animals’ whole bodies caused a slowing response similar to the one observed upon food entry as reported previously^25^ (Fig. 4b). The effect was observed only in the presence of all-trans-retinal (ATR), which should be supplied as a co-factor of ChR2 for the animals^40^. We then illuminated the blue light to a specific pair of DAergic neurons with the optogenetic setup of the OSB system (Fig. 2b). When the CEPD pair was specifically activated, a significant slowing response was observed, whereas CEPV activation did not cause significant slowing (Fig. 4b). We also tested PDE pair but observed no response. These results indicate that CEPD pair activation is mostly responsible for the slowing behaviour.

## Discussion

Here we revealed the functional diversification of DAergic neurons in *C. elegans*. To analyse the physiological properties of DAergic neurons that are activated by the mechanical stimulus of bacterial lawn, we established an integrated microscope system for *in actio* optophysiological analyses and found that CEPD and CEPV neuron pairs, which have been considered highly similar to each other based on their anatomical symmetry and similar gene expression patterns, are functionally asymmetrical in terms of their responsiveness to the bacterial lawn stimulus and the effect of their activation on the slowing response.

To individually analyse the food entry–dependent activation patterns of DAergic neuron pairs of *C. elegans*, which are located in close proximity and cannot be separated with known cell-specific promoters, we established the OSB system, which tracks freely-moving animals with high spatiotemporal resolutions and performs calcium imaging and optogenetic illumination of individual neuron pairs. Optogenetic illumination requires higher specifications than calcium imaging. For calcium imaging, multiple neuronal activities can be simultaneously captured during tracking and analysed individually afterward (*i.e*., off-line): individual neurons do not require separate identification during tracking. In contrast, for optogenetic illumination, individual neurons should be identified during tracking (*i.e*., on-line). For temporal resolution, the widely used optogenetic microscope systems for moving *C. elegans* track an animal with a cycle speed of 25–50 Hz^29^,^30^, while our system does the same at 200 Hz. If a system possesses a spatial resolution of 2–3 μm (the regular diameter of the animal’s neuronal cell body^41^), an animal moving at 150 μm/s should be tracked at >50 Hz (for details, see Stirman *et al*.^30^). For spatial resolutions, the aforementioned two systems use a 4–10× objective lens to capture the image of the animal’s entire body for tracking^29^,^30^, while our system uses a 20× or higher objective lens because it allows tracking of a part of the animal’s body by using a high-speed pattern-matching algorithm^34^. The two systems have been used in many laboratories for individual optogenetic analyses of multiple neurons and/or muscles that are sufficiently separated, such as those in the middle and posterior parts or on opposite sides of the body^29^,^30^,^42^,^43^,^44^, but not for multiple neurons within the animal’s brain. While there are a few other systems for calcium imaging or optogenetic analyses of multiple neurons in the brain of the animals^27^,^28^,^31^,^32^, our OSB system allows for both calcium imaging and optogenetic analysis and revealed the functional asymmetry of DAergic neuron pairs of moving *C. elegans*, which are separated by only 20–25 μm in the brain. Our system can also be used for functional dissection of other neurons that are genetically indistinguishable and located in close proximity; it could also be used with spatially and temporally more complex pattern of optogenetic illuminations to reveal dynamic relationships of activities of multiple neurons in future experiments.

We found that the CEPD and CEPV neurons are both tonically activated. Sensory neurons generally exhibit phasic, tonic or phasi-tonic responses^45^. Phasic response signals the changes in stimulus over a wide range, which contributes to an animal’s navigation in a chemical gradient, for example. The tonic response signals the presence/absence and strength of the stimulus over time. Tonic signalling is used to provide positional information about the body parts (proprioception) or for biologically significant chemicals such as CO_2_^45^. These functions of phasic and tonic signalling are also true in *C. elegans*. Sensory neurons for chemotaxis or thermotaxis show phasic responses^46^,^47^,^48^, while those for sensing O_2_ show tonic responses^49^. As such, it is reasonable to speculate that the *C. elegans* DAergic neurons are tonically activated to signal the information of food presence, which modulates various aspects of the animal’s behaviour. In a previous study, CEP neurons exhibited a rapidly-adapting (*i.e.*, phasic) response to a mechanical press with a glass probe^50^, suggesting that the tonic response observed in this study might be triggered by a subtle and/or fluctuating mechanical pressure brought about by the animal’s movement on a bacterial lawn. Quite interestingly, tonic signalling of DA is well known in primates and rodents: Tonic DA release enables a variety of motor, cognitive, and motivational processes, while phasic DA release signals rewards and alerting stimuli^3^. Thus, the role and mechanism of tonic DA signalling in the modulation of various aspects of the nervous system may be evolutionarily conserved from nematodes to mammals.

Our analysis revealed that CEPD and CEPV pairs are functionally asymmetrical. Not only were their activation patterns different, but the effects of their optogenetic activation differed: In both cases, CEPD played a more significant role. In *C. elegans*, there are a few precedent examples of the asymmetrical functions of anatomically symmetrical sensory neurons, including ASEL-ASER^48^ and AWC^ON^-AWC^OFF^ (ref. 51). These functional asymmetries are related to an increased repertoire of the sensory stimuli to which each sensory neuron can respond. This is likely because the number of sensory neurons is quite limited in the animals (only a few pairs of sensory neurons are used to detect tens of odorants, for example^17^). This may also be the case for CEP neuron pairs.

The functional asymmetry of CEP neuron pairs was unexpected because not only do they appear to be anatomically symmetrical, but DA is known to function extrasynaptically to motor^39^ and sensory^12^,^25^ neurons. The fact that optogenetic CEPV activation did not cause the slowing response may suggest that, at least for that particular behavioural modulation, DA function is mediated intrasynaptically. CEP neurons have 77 known postsynaptic target neurons, of which 14 neurons have synaptic inputs from both CEPD and CEPV neurons (“DV” in Table S1 according to http://wormwiring.hpc.einstein.yu.edu). Most of the remaining synaptic connections are from only one of CEP neurons (“single” in Table S1). Thus, the unshared synaptic connections from CEPD may play a significant role in the slowing response. Alternatively, the different amounts of released DA caused by similar optogenetic activations could be the reason for the different slowing response between CEPD and CEPV pairs; even in that case, DA released by CEPV is likely to have its own role.

We also found that another DAergic neuron pair, the PDEs, were not activated upon food entry and did not cause slowing with optogenetic activation. Although all of the DA pairs are considered functionally redundant, laser ablation of CEPs, but not ADEs or PDEs, significantly affected the slowing response^8^. Similarly, CEPs, but not ADEs or PDEs, have been recently reported to be responsible for learning of food patch size^52^. In addition, only CEP neuron pairs have their sensory endings at the anterior end of the body (Fig. 1a). Taken together, these data suggest that DA from CEPD plays a major role in the slowing response, while DA from other DAergic neuron pairs has different roles but still plays a minor role in the slowing response, possibly through extrasynaptic transmission. The other pairs may be activated in a much longer time-scale in the presence of food, by different stimuli, or in different contexts.

So where do the differences in DAergic neuron pairs originate? The identities of neuronal types are determined by the expression of type-specific batteries of genes, such as the ones for biosynthesis of neurotransmitters, ion channels, neurotransmitter receptors, and signalling molecules^2^. Such identity of DAergic neurons is tightly regulated by three types of transcription factors in *C. elegans*, as well as in mammals^19^,^20^. Nevertheless, the CEP pairs are functionally asymmetrical and have different synaptic partners. Thus, our results suggest there are yet-unknown mechanisms that could further determine the differences in the same neuron type beyond genetic terminal identification.

## Methods

### Strains

The techniques used to culture and handle *C. elegans* were essentially as described previously^53^. The *C. elegans* wild-type Bristol strain (N2) and the mutant strains CB1112 *cat-2(e1112)*, TQ1101 *lite-1(xu7)*, and TQ296 *trp-4(sy695)* were obtained from the Caenorhabditis Genetics Center (University of Minnesota, USA). Young adult hermaphrodites were used in all of the behavioural experiments.

### Behavioural assay for bending numbers

For the food patch, the OP50 bacteria was grown in 100 mL of LB culture overnight, spun down, and suspended in 10 volumes of nematode growth medium (NGM) buffer, and 5 μL of the suspension was applied to four spots 5 mm from the centre of the NGM plate surface (Fig. 1b); this bacterial lawn was 30–40 μm thick and did not obstruct the fluorescent imaging. Five young adult worms were briefly washed with 10 μL of NGM buffer and placed on 1.7% agar NGM plates with food patches. The worms moved freely and spontaneously entered the food during video recording with a dissection microscopy SZX10 and a digital camera system DP72 (Olympus). The obtained images were manually analysed to quantify bending number as described previously^8^.

### Molecular biology and germline transformation

For the DAergic neuron–specific expression, mCherry^36^, GCaMP6f 35, and codon-optimized ChR2(H134R)^54^ cDNAs were fused with *dat-1* promoter^55^ using a GATEWAY system^®^ (Thermo Fisher Scientific). ChR2 as well as mCherry were expressed in *lite-1(xu7)* mutant background to reduce the animal’s response to strong blue light^56^. Germline transformation was performed using microinjection^57^. Multiple transgenic lines were used for each experiment type, and the different lines produced similar results. The plasmids and transgenic lines used in this study are listed in Supplementary Tables S2 and S3, respectively.

### Confocal imaging

The animals were anesthetized with 5 mM sodium azide on a 2% agarose NGM pad and covered with a cover glass (Matsunami). Images of the animals expressing *dat-1*p::mCherry and *dat-1*p::ChR2::GFP were captured with a confocal microscope LSM710 (Carl Zeiss) with a Plan Apochromat 40× 0.95 NA lens. The images were processed using Zen software (Carl Zeiss).

### Behavioural tracking

An animal on an NGM plate with food patches was placed on a motorized stage HV-STU-03W (HawkVision Inc., Fujisawa, Japan) combined with a BX51WI upright microscope (Olympus). The animal was illuminated with infrared (IR) light from a halogen lamp without an IR-cut filter through a 32BP775 band-pass filter (Olympus), and the bright field image was reflected with a DF670 dichroic mirror (Semrock, USA) guided to a GRAS-03K2M-C CCD camera (Point Grey Research, Canada) with a 0.35× U-TV0.35XC-2 C-mount TV adapter (Olympus). The images were taken at 200 Hz by the camera and processed by a custom-made program for real-time pattern matching on a Linux PC (Intel Core i7-870) that regulated the motorized stage to maintain the region of interest (ROI) of a freely-moving animal in the centre of the microscope’s field of view^34^. In most of the frames, a ROI was set around the head ganglia. For PDE imaging, the ROI was set around the fluorescence of PDE cell body. Each animal’s speed was calculated from records of displacement of the auto-tracking stage and ROI position in the field of view.

### Calcium imaging

The sample was exposed to white excitation light from a Multi-independent Light Stimulation System (MiLSS; Aska Company, Hyogo, Japan)^37^ through a BP460-495 bandpass-filter (Olympus) and a DM505HQ dichroic mirror (Olympus). The fluorescence was introduced into a W-View optics system (Hamamatsu, Japan) containing two dichroic mirrors (545SP and 545LP) and bandpass filters (D520/20m and 620DF35 for GCaMP and mCherry, respectively) through a 20× UPLFLN objective lens (Olympus). The GCaMP and mCherry images were split by the W-View and simultaneously captured side-by-side on an EM-CCD camera ImagEM (Hamamatsu). Images were taken at a 32.6-ms exposure time and 100-ms sampling interval with 2  × 2 binning. The calcium imaging subsystem was triggered by a TTL signal from the auto-tracking subsystem, and both subsystems ran independently thereafter in the present setup. The images were saved as uncompressed 16-bit TIFF files, the cell bodies were independently tracked with a custom-made off-line optical flow tracking program, and signal intensities of particular regions were measured by ImageJ (NIH). The signal intensity of the background was subtracted from that of the cell body. This process was performed for fluorescent images from both green (GCaMP) and red (mCherry) channels of W-View, and the ratio of fluorescent intensities of GCaMP over mCherry (*R*) was calculated to cancel out florescent changes caused by motion artefacts. The median ratio during 90 seconds before the food entry was defined as the baseline *R*_*0*_, and the normalized ratio (*R/R*_*0*_) was then calculated and further analysed.

### Optogenetic experiments

The animals were grown in the presence or absence of ATR as described previously^58^ and transferred to an NGM plate on the OSB system and maintained under the 20× objective lens by auto-tracking. The sample was illuminated with green and blue lights with MiLSS through an XF3069 multi-bandpass filter (Omega) and an FF493/574-Di01 dichroic mirror (Semrock). The green light (540–570 nm; ~0.4 mW/mm^2^) was illuminated to the full view field for the real-time optical flow tracking of the mCherry-expressing DAergic neuron positions. The fluorescent images of mCherry were captured through the 620DF35 bandpass filter. Each DAergic neuron was identified based on vulva position from the bright-field images, and one was manually selected and then automatically targeted by the optical flow tracking. After 1 min for measuring basal locomotion rate and when the worm was moving forward, blue light (450–470 nm; ~12 mW/mm^2^) was overlaid for 10 s to the full view field or at the position of targeted cell body, which was updated at 31.7 Hz; because the motorized stage adjusted the position of the animal’s brain at 200 Hz, the relative neuron speed was slow enough for single cell targeting at 31.7 Hz. A locomotion speed of 10 s before and during stimulation was calculated according to the behavioural tracking of the animals and normalized with the ensemble median of averaged locomotion speed before stimulation for each targeted cell type. One animal was tested only once for the light stimulation to avoid any hysteretic effects.

To verify the spatiotemporal accuracy of our cell-targeted light stimulation, we first illuminated the full view field with green light at ~0.4 mW/mm^2^ to visualize all of the mCherry-expressing neuronal cell bodies and then targeted one of the cell bodies with green light at ~2.6 mW/mm^2^ to further excite the mCherry fluorescence on that cell only. The results (Video S3) show that the targeted light illuminated the target cell body but not any others.

### Data analysis and statistics

The data were obtained over 3 days from 10–40 animals for each condition in most cases. We chose this sample number because a large-scale behavioural analysis of *C. elegans* concluded that a certain statistical difference can be detected in 10 animals, and 24 ± 14 (average ± SD) animals per condition were used^59^. After the sample acquisition, the data of some animals for Figs 3 and 4 were excluded when any of the following problems were found: (1) trials interrupted by auto-tracking errors, (2) insufficient mCherry or basal GCaMP6f fluorescence intensity for optical flow tracking, or (3) basal locomotion speed <0.06 mm/s (average speed ± SD of normal animals was 0.17 ± 0.05 mm/s). In addition, when the average of *R/R*_*0*_ after *t* = 0 was <1.1 in an animal’s CEPD and CEPV, the animal was regarded as “not responding”; those data were not included in Fig. 3 but are shown in Table 1.

Experimental conditions, such as the presence/absence of ATR, light stimulation, or different strains, were randomized on a daily basis. A Kruskal-Wallis test with a *post hoc* Steel-Dwass test was used for multiple comparisons in Fig. 4b, while a Mann-Whitney test was used for single comparison in Figs 3c and 4b using R (The R Project) or Prism ver. 5.0 for Mac OSX (GraphPad Software, San Diego, CA, USA).

## Additional Information

**How to cite this article**: Tanimoto, Y. *et al*. *In actio* optophysiological analyses reveal functional diversification of dopaminergic neurons in the nematode *C. elegans*. *Sci. Rep*. **6**, 26297; doi: 10.1038/srep26297 (2016).

## Supplementary Material

Supplementary Information

Supplementary Video S1

Supplementary Video S2

Supplementary Video S3

## Figures and Tables

**Table 1:** Numbers of animals that exhibited calcium response to food entry.

Strain	Feeding status	Cell	# Tested	# Responded	% Responded
Wild-type	fed	CEPD, CEPV	n = 39	n = 28	71.8%
Wild-type	starved	CEPD, CEPV	n = 16	n = 11	68.8%
Wild-type	fed	PDE	n = 26	n = 6	23.1%
*trp-4(sy695)*	fed	CEPD, CEPV	n = 8	n = 3	37.5%

When the average *R/R0* ≥ 1.1 in either CEPD or CEPV cells of an animal after food entry, the animal was scored as “responded”.
