# Supplementary material for: In actio optophysiological analyses reveal functional diversification of dopaminergic neurons in the nematode C. elegans
Source: Sci Rep. 2016 May 19;6:26297. doi: 10.1038/srep26297 (PMC4872038; doi:10.1038/srep26297)
Supplement: Supplementary Information [file srep26297-s1.pdf]

Supplementary information

Title:

In actio optophysiological analyses reveal functional diversification of dopaminergic neurons in the nematode *C. elegans*

Authors: Yuki Tanimoto[1]*, Ying Grace Zheng[1]*, Xianfeng Fei[2], Yukako Fujie[1], Koichi Hashimoto[3] , Koutarou D. Kimura[1]

Items:

Supplementary Figure S1

Supplementary Table S1 - S4

Supplementary Video S1 - S3

Wilde-type, well-fed, PDE

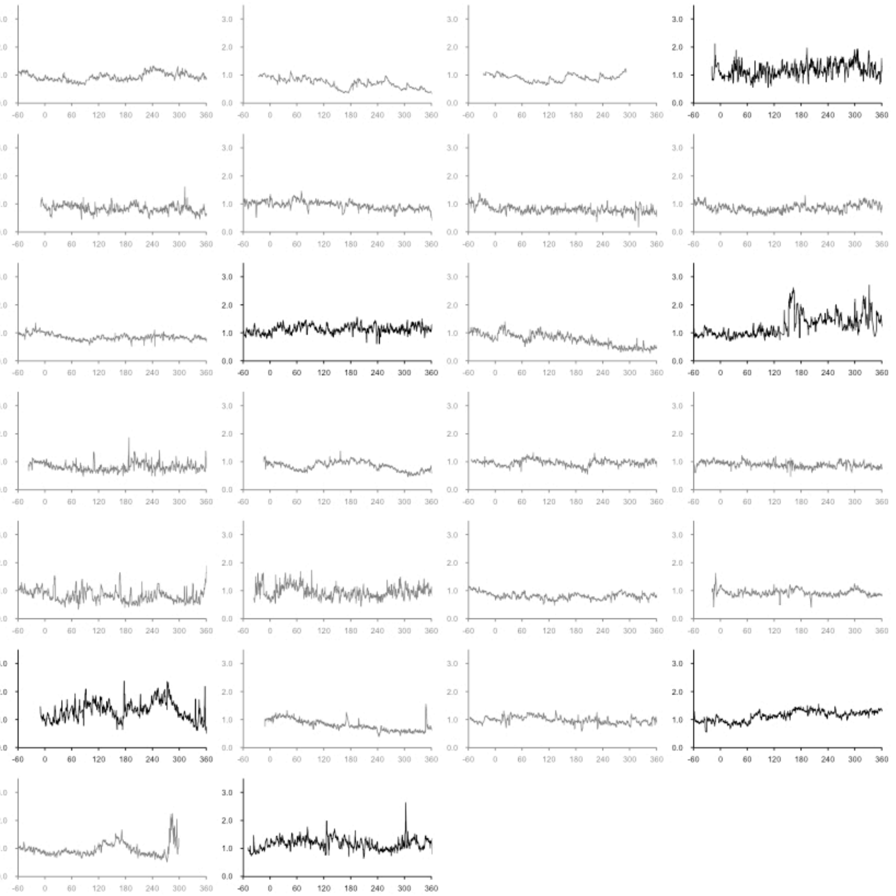

trp-4(sy695), well-fed, CEPD

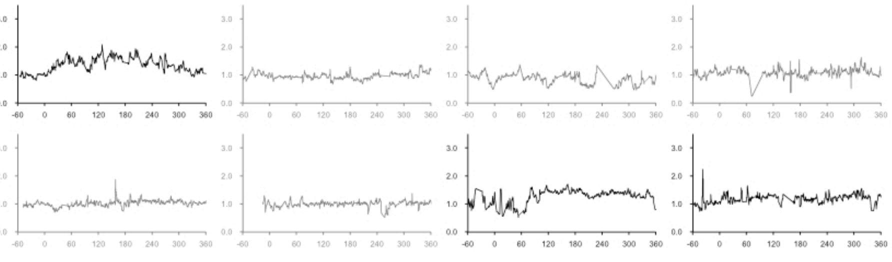

trp-4(sy695), well-fed, CEPV

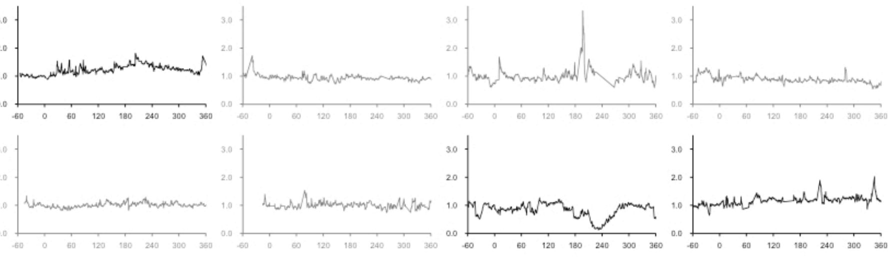

Figure S1. Individual calcium responses of PDE pair from well-fed wild-type animals and CEPD and CEPV neuron pairs from well-fed *trp-4* animals. When the average R/R_0 after $t = 0$ was <1.1 in PDE or in both of CEPD and CEPV in an animal, the animal was regarded as "not responding" one and the data is shown in grey. Similarly to Fig. 3, the time when an animal entered a bacterial lawn was determined as $t = 0$.

Supplementary Table S1. Numbers of synaptic connections from CEP neurons to target neurons.

Targeted neuron	CEPDL	CEPDR	CEPVL	CEPVR	Targeting CEP
ADLR				2	single
AFDL	1				single
AINL	1				single
ALML	2				single
ASGR				2	single
AVEL		6		7	DV
AVER	8		3		DV
BDUR		3			single
CEPDR	1	3		1	DV and LR
CEPshVL			1		single
CEPshVR				2	single
IL1R		1			single
IL1DL	6				single
IL1DR		7			single
IL1VL			2		single
IL1VR				4	single
IL2DL	1				single
IL2VR				1	single
OLLL	4		3		DV
OLLR		7		8	DV
OLQDL	8				single
OLQDR		9			single
OLQVL			5		single
OLQVR				6	single
PVR			1		single
RIAL			1	1	LR
RIAR				1	single
RIBL	2		1		DV
RIBR		1			single
RICL	7	8	6	3	DV and LR
RICR	4	9	4	3	DV and LR
RIFR		1			single
RIH	1	1			LR
RIPL	3		2		DV
RIPR				1	single
RIS	2				single
RIVL				1	single
RMDR		1			single
RMDDL	1		6		DV
RMDDR				3	single
RMDVL	9				single
RMDVR		2	1		DV
RMGL	4		1		DV
RMGR		1			single
RMHL	1	3	3		DV and LR
RMHR	6	1	4	4	DV and LR

(continue)

(continued)

Targeted neuron	CEPDL	CEPDR	CEPVL	CEPVR	Targeting CEP
SIADR	2				single
SIAVL			1		single
SIAVR				4	single
SIBDR	1				single
SIBVR		1			single
SMBDL		1			single
SMBDR	3				single
SMBVL			1		single
SMBVR				1	single
SMDDR				1	single
SMDVR			1		single
URADL	3				single
URADR		2			single
URAVL			2		single
URAVR				2	single
URBL	6				single
URBR		7			single
URXL			1		single
URXR		2			single
URYDL	3				single
URYDR		1			single
URYVL			3		single
URYVR				1	single
dBWMR3		3			single
dBWML5	1				single
dBWMR5		2			single
vBWMR3				1	single
vBWML5			1		single
vBWMR5				2	single
vBWML7			2		single
vBWMR8				2	single
glial			1		single

CEPDL, CEPD-left; CEPDR, CEPD-right; CEPVL, CEPV-left; CEPVR, CEPV-right.

Supplementary Table S2. Plasmids generated for this study.

Plasmid name	Promoter	cDNA
pYFU207	*dat-1*[55]	mCherry[36]
pYFU210	same as above	GCaMP6f[35]
pYFU230	same as above	ChR2(H134R)::GFP[54]

Supplementary Table S3. Transgenic strains used in this study.

Fig. no.	strain name	genotype	DNA injected
Fig. 3	KDK53204<br>KDK53214<br>KDK53236<br>KDK53250	*N2;oskEx53204*<br>*N2;oskEx53214*<br>*N2;oskEx53236*<br>*N2;oskEx53250*	45 ng/μl of *dat-1p::GCaMP6f* (pYFU210)<br>45 ng/μl of *dat-1p::mCherry* (pYFU207)<br>10 ng/μl of *lin-44p::mRFP* (a gift from Dr. M. Koga)<br>10 ng/μl of PvuII-cut N2 genome as a carrier
Fig. S1	KDK53289	*trp-4(sy695);oskEx53204*	same as above (the extrachromosomal array was transferred from KDK53204 by mating)
Fig. S1	KDK53291	*trp-4(sy695);oskEx53236*	same as above (the extrachromosomal array was transferred from KDK53236 by mating)
Fig. S1	KDK53293	*trp-4(sy695);oskEx53250*	same as above (the extrachromosomal array was transferred from KDK53250 by mating)
Fig. S1	KDK53295	*trp-4(sy695);oskEx53214*	same as above (the extrachromosomal array was transferred from KDK53214 by mating)
Fig. 4b	KDK53665<br>KDK53715	*lite-1(xu7);oskEx53665*<br>*lite-1(xu7);oskEx53665*	60 ng/μl of *dat-1p::ChR2(H134R)::GFP* (pYFU230)<br>20 ng/μl of *dat-1p::mCherry* (pYFU207)<br>20 ng/μl of PvuII-cut N2 genome as a carrier

Supplementary Table S4. Details of the statistical analyses shown in Fig. 4b.

Multiple comparison results
Statistical test: Kruskal-Wallis
Comparison of interest: Normalized locomotion speeds of each condition in the presence of blue light illumination
P value: 0.0002

***Post hoc* Steel-Dwass test results with blue light**

Comparison	*P* value
All (ATR -) vs All (ATR +)	0.0019
All (ATR -) vs CEPD (ATR +)	0.0053
All (ATR -) vs CEPV (ATR +)	0.2744
All (ATR -) vs PDE (ATR +)	0.6299

Two-tailed Mann-Whitney test results with or without blue light

Targeted cell type	*P* value
All (ATR -)	0.4307
All (ATR +)	0.0043
CEPD (ATR +)	0.0185
CEPV (ATR +)	0.2150
PDE (ATR +)	0.5972

Supplementary Video Legends

Supplementary Video 1. Tracking of dopaminergic neurons. GCaMP fluorescence (left) and Bright field image (right) are shown. The video speed is 1×.

Supplementary Video 2. Off-line optical flow tracking of fluorescent signals from multiple DAergic neuronal cell bodies for calcium imaging.

Supplementary Video 3. On-line optical flow tracking of fluorescent signals from multiple dopaminergic neuronal cell bodies for optogenetic stimulation. One of CEP neuron was targeted.
